# A conversation with Andrew H. Talal, MD, MPH, professor of medicine, university at buffalo jacobs school of medicine and biomedical sciences

**DOI:** 10.1017/cts.2025.10095

**Published:** 2025-08-13

**Affiliations:** Clinical Research Forum, Washington, DC, USA

## Top 10 clinical research achievement awards Q & A

This article is part of a series of interviews with recipients of Clinical Research Forum’s Top 10 Clinical Research Achievement Awards. This interview is with Andrew H. Talal, MD, MPH, Professor of Medicine, University at Buffalo Jacobs School of Medicine and Biomedical Sciences. Dr Talal’s research focuses on improvements to the treatment of patients with viral hepatitis and other forms of liver disease. He received the 2025 Distinguished Clinical Research Achievement Award for “Hepatitis C Treatment Through Facilitated Telehealth in Opioid Use Disorder.” *The interview has been edited for length and clarity.*

## How did you get your start in clinical research?

My career in clinical research started when I was a fellow in gastroenterology at the University of North Carolina Chapel Hill. I had finished medical school at the University of Texas at San Antonio and residency at the University of Iowa. The University of North Carolina at Chapel Hill had an National Institutes of Health (NIH) supported research training program that allowed me to earn a master’s in public health (MPH) at the same time that I completed my clinical training in Gastroenterology. I was interested in viral infections, so as part of my master’s thesis, I worked on a project that showed how Norwalk agent – or as it’s now called, norovirus – is an important cause of infantile nausea and vomiting.

## Why did you pursue an MPH after becoming an MD?

The MPH helped me learn the tools needed for integrating a population-based approach with translational research. My father was a rheumatologist who conducted laboratory based research, and I could see how the techniques that were becoming available in the 1990s – Polymerase Chain Reaction (PCR), genetic microarray, and others – enabled us to ask clinical questions in new ways. These tools led to new perspectives and insights.

## What came next on your career path?

After I finished my fellowship, I worked with Dr David Ho, the renowned researcher and physician who has contributed to understanding and treating Human Immunodeficiency Virus (HIV) infection. I arrived in his lab just as he was awarded *Time* magazine’s “Man of the Year” in 1996. While under his mentorship, I researched HIV in the gastrointestinal-associated lymphoid tissue. In 2000, I became an Assistant Professor at Weill Cornell Medicine where I focused on research and clinical care of hepatitis C virus infection.

## All of this came together, leading to the award-winning research?

Yes, for the first 15 years of my career, I led a laboratory doing translational research. We investigated biomarkers of hepatitis C infection and used liver biopsies to measure drug distribution and concentration in the liver during antiviral treatment. But around 2010–11, it became clear that there was a major public health issue broadly concerning the limited access to hepatitis C virus treatment and especially in methadone programs. Hepatitis C is a common condition in people who have opioid use disorder because of shared needles. In New York State where people go in person up to six or seven days a week to receive methadone, they weren’t going to receive treatment for hepatitis C virus infection if they had to be referred off-site. Hepatitis C is very common in people with opioid use disorder because of sharing of contaminated needles. A therapeutic divide existed since hepatitis specialists were not going to methadone programs and vice versa. To address the access gap, I wondered if we could use digital technology, specifically telemedicine, to bring the doctor to the patient in the methadone program. Keep in mind, this was pre-COVID, so telemedicine use was much less commonplace.

## What is the standard of care for hepatitis C?

In 2010–11, hepatitis C was treated with interferon, a cancer chemotherapeutic agent, given by injection. But interferon can make people feel terrible and exacerbate mental health issues such as depression and anxiety, which many people with opioid use disorder are already struggling with. Fortunately, around this same time, 2012–13, treatment for hepatitis C was shifting to direct-acting antiviral (DAA) medications. DAAs are administered as pills and they have tremendous effectiveness, with minimal side effects. They can achieve a cure within 2 to 3 months. Once DAAs were available, we were able to run a pilot study with 45 patients and show that 93% of patients could be cured through facilitated telemedicine.

## What did the award-winning research show?

The study included 602 participants, and it demonstrated that opioid treatment program-integrated facilitated telemedicine resulted in significantly higher hepatitis C virus intention-to-treat cure rates (90.3%) compared with off-site referral (39.4.%), with high participant satisfaction. In addition, illicit drug use declined significantly among cured participants in both study arms with minimal reinfections over two-years of follow up.

## Why is hepatitis C infection such an effective use case for this kind of study?

Several factors promote hepatitis C as an excellent use case for a study of treatment access among an underserved population. In the vast majority of cases, hepatitis C virus infection can be cured if people adhere to treatment as prescribed. Additionally, short treatment duration and the fact that a cure is a biologic outcome, that is, yes or no, you either have the virus or you do not. Factors like those help make hepatitis C infection a great use case to begin to think about how technologies, like telemedicine, can be used to improve treatment access for underserved populations.

## How was telemedicine integrated into hepatitis C management for this trial?

We used a facilitated telemedicine model, so there was a case manager on site at each of the methadone programs. They did all the care coordination and served as a facilitator, becoming a conduit and a disseminator of trust to the patients. When we started this work with the pilot study, we weren’t sure if someone in substance use treatment would trust a doctor they were interacting with through a camera. We found, through interviews with patients and staff in these opioid treatment programs, that the patients felt they had an opportunity to form a collaboration with the case manager. The case managers did the warm handoffs, the introductions between the patient and the physician as well as facilitating the telemedicine interactions. Not only did the patients appreciate this, but the staff felt this intervention allowed them to help take care of a disease that is really not within the scope of their typical program. They were able to connect the people in their methadone clinics with hepatitis C champions.

## How did the study teams stay connected over the course of the trial?

We had weekly meetings, and in the years when we were actively recruiting patients, we tried to visit every site every year. At the time, hepatitis C treatment was shifting to DAAs so we would have learning lunches where we would provide lunch (or breakfast) for the staff and use that time to review protocols. It was a fantastic way to educate people about changes that were happening in the field and to talk to them about our study. We also used – and I must thank Dr Marianthi Markatou, the Head of Biostatistics and Data Science Unit of the trial for this – the stepped wedge design, so that each of the 12 participating methadone clinics was randomly assigned to switch to the telemedicine intervention during different study periods. The annual visits allowed us to visit the sites before they initiated telemedicine, work through any concerns, and course correct as needed. For instance, one site was concerned about people seeing others getting extra medication at the methadone dispensing window, so we were able to increase privacy there.

## Where is this research headed next?

We are interested in pursuing how to implement facilitated telemedicine in other settings, such as rural primary care and different types of community-based prevention interventions like syringe service programs. Typically, these have a much more transient population than participated in the methadone programs in our study.

## What advice would you give to someone starting their career in clinical research?

My first piece of advice is to think very carefully and creatively about what you want to study. When you do, you may find unique opportunities, particularly in areas where you might not have seen them before. Secondly, you need to choose excellent partners – on your team, as mentors, and with institutions. You want to work with people and be in places where you’re supported, at all levels. Clinical research takes time and there are always going to be challenges. It’s essential that you know what’s important to you and that you stay creative in terms of how you go about getting what you need to be successful.

